# Real-world experience with foslevodopa/foscarbidopa in Germany: therapeutic insights and lessons learned

**DOI:** 10.1186/s42466-026-00530-3

**Published:** 2026-08-31

**Authors:** David Weise, Tobias Warnecke, Carsten Buhmann, Georg Ebersbach, Carsten Eggers, Björn Falkenburger, Günter Höglinger, Brit Mollenhauer, David Pedrosa, Martin Südmeyer, Daniel Weiss, Jan Kassubek, Wolfgang H. Jost

**Affiliations:** 1 Department of Neurology, Asklepios Fachklinikum Stadtroda, Stadtroda, Germany; 2 Department of Neurology, University of Leipzig, Leipzig, Germany; 3 Department of Neurology and Neurorehabilitation, Klinikum Osnabrück - Academic Teaching Hospital of the University of Münster, Osnabrück, Germany; 4 Department of Neurology, University Medical Center Hamburg-Eppendorf, Hamburg, Germany; 5 Movement Disorders Hospital Beelitz, Beelitz-Heilstätten, Germany; 6 Department of Neurology, Knappschaft Kliniken Bottrop, Bottrop, Germany; 7 Department of Neurology, Faculty of Medicine, University Hospital Carl Gustav Carus, Technische Universität Dresden, Dresden, Germany; 8 Department of Neurology, LMU University Hospital, Ludwig-Maximilians-Universität (LMU) München, Munich, Germany; 9 Department of Neurology, Universitätsmedizin Göttingen, and Paracelsus-Elena- Klinik, Kassel, both Germany; 10 Philipps-University Marburg, Marburg, Germany; 11 Department of Neurology, Klinikum Ernst von Bergmann, Potsdam, Germany; 12 Department of Neurology, University Medical Center Düsseldorf, Düsseldorf, Germany; 13 Centre for Neurology, and Hertie-Institute for Clinical Brain Research, University of Tübingen, Tübingen, Germany; 14 Department of Neurology, University Hospital Ulm, Ulm, Germany; 15 Parkinson-Klinik Ortenau, Wolfach, Germany

**Keywords:** Parkinson’s disease, Foslevodopa/Foscarbidopa, Real-world experience, Recommendations, Device-aided therapy

## Abstract

Patients with advanced Parkinson’s disease (PD) often need to continue with device-aided therapies (DAT), such as intestinal and subcutaneous infusion therapies. Recently, continuous subcutaneous administration of foslevodopa/foscarbidopa (LDp/CDp) has emerged as a new therapeutic method. Two years after its approval in Germany, a large amount of real-world experience has been collected. Based on this, we present important practical recommendations to help clinicians optimise treatment. Key topics include identifying suitable patients, approaches to initiation and dose determination as well as strategies for balancing LDp/CDp with concomitant oral dopaminergic therapies. Practical challenges, particularly infusion site reactions and neuropsychiatric vulnerability in older or cognitively impaired patients, are discussed, and recommendations are presented on how to prevent and manage these adverse effects during routine management. The review also addresses reasons for discontinuation, organisational factors relevant to real-world implementation and future directions in pump technology. Overall, LDp/CDp is effective in managing motor fluctuations and provides sustained benefits with an overall favourable tolerability profile. Dermatological reactions are common but manageable, and careful adjustment of oral medication rather than rapid pursuit of monotherapy is advisable. The rate of early discontinuation has decreased with growing clinical experience.

## Background

Parkinson’s disease (PD) is the second most common neurodegenerative disorder in elderly people. The prevalence, disability and mortality associated with PD have more than doubled over the past generation [1]. This trend is expected to continue [2], highlighting the need for better prevention and treatment strategies.

Oral levodopa is the main treatment for PD [3, 4]. However, the effectiveness can decline over time, leading to the development of motor and non-motor fluctuations. A marker of these fluctuations are ‘OFF’ periods, when the effects of dopaminergic treatment wear off and motor symptoms, such as bradykinesia, rigidity and tremor, re-emerge or worsen. These episodes may also include non-motor symptoms such as anxiety, pain as well as cognitive changes and can significantly impair patients’ quality of life [5]. Over the course of PD, motor and non-motor fluctuations happen regularly and are steadily increasing as the synaptic degeneration progresses: after five years, nearly 50% of patients are affected, increasing to nearly all patients after ten years [6]. Despite treatment options available to manage these fluctuations by optimising oral dopaminergic therapy, many patients eventually require a transition to device-aided therapies (DAT). These include intestinal and subcutaneous infusion treatments as well as deep brain stimulation (DBS). Recent guidelines recommend introducing these DAT to improve motor and non-motor symptom control and quality of life [7, 8]. Furthermore, earlier DAT introduction might prevent neuropsychiatric complications such as hallucinations, impulse control disorders (ICD) or dopamine dysregulation syndrome, however, it has to be noted that the advantages of earlier introduction have been demonstrated for DBS [9], but will need confirmation by randomised controlled trials for pumps. With more DAT becoming available, the treatment landscape for advanced PD is becoming increasingly individualised [10, 11].

Continuous subcutaneous infusion of foslevodopa/foscarbidopa (LDp/CDp) is now available as a new DAT. This medication contains prodrugs of levodopa and carbidopa, delivered continuously via a usually 24-hour subcutaneous infusion using a portable pump. The steady delivery helps to maintain consistent levodopa blood levels that seem to correlate with stable striatal dopamine levels [12], reducing fluctuations. The first phase 3 trial, a 12-week long study comparing LDp/CDp to oral levodopa, showed a significant increase in ‘ON’ time without troublesome dyskinesia and a significant reduction in OFF time [13]. These changes were maintained over 52 weeks, and furthermore, the prevalence of morning akinesia dropped from 77.7% at baseline to 27.8% by week 52 and sleep quality as well as overall quality of life also improved [14, 15]. 

In December 2023, Germany became the second country after Japan to approve continuous subcutaneous infusion of LDp/CDp. To date, Germany has been the country where the highest number of patients worldwide have started this therapy. Hence, major clues can be drawn from this experience in the early adoption of this treatment, providing valuable insights into patient selection, implementation and long-term outcomes in routine clinical practice. A key feature is that initiation of LDp/CDp therapy typically occurs in an inpatient setting in Germany, often as part of multimodal complex therapy programmes [16–18], but sufficient initiation in a day-clinic setting has also been reported [19]. The core requirement is the presence of structures allowing for optimal evaluation, individualised titration and multidisciplinary support. Based on two years of real-world LDp/CDp use, this manuscript outlines important practical lessons to help clinicians optimise treatment and complements existing reviews, which have largely focused on clinical trial data, by providing additional insights from routine clinical practice. Building on existing recommendations, including those incorporating clinical experience, it offers further practical insights into treatment initiation, dose optimisation, and the management of LDp/CDp in everyday care, including safety considerations.

## Patient profiles

LDp/CDp is indicated for treatment of advanced levodopa-responsive PD with severe motor fluctuations and hyperkinesia/dyskinesia when combinations of oral or transdermal dopaminergic treatment did not provide satisfactory results anymore. Identifying suitable patients for advanced therapies in routine clinical practice has remained challenging. The ‘5-2-1 rule’ [20] is a screening tool in outpatient settings to support this process: suspected indicators for advanced PD are five or more daily doses of oral levodopa, at least two hours of OFF time per day or at least one hour of troublesome dyskinesia. Meeting at least one of these criteria should encourage referral to a specialist for further evaluation.

DAT, such as DBS and pump therapies, can be beneficial for patients with advanced PD with severe response fluctuations despite optimised treatments [8]. However, real-world data suggests that DAT are often introduced late, with many patients either refusing these therapies feeling that DAT was not yet necessary or needing more time to decide [21–23] or fear the risk of invasive therapies such as DBS or intestinal infusions. Such delays carry the risk of missing a window of opportunity when patients still have functional independence and greater cognitive ability. By contrast, LDp/CDp is a non-surgical method that is comparatively convenient and can lower the threshold for starting continuous therapy among both patients and clinicians. A post-hoc analysis of a small group (*n* = 26) of younger patients (aged ≤ 65 years) at an earlier stage of advanced PD (defined as being within five years of experiencing motor fluctuations, with a Hoehn & Yahr stage of ≤ 2 in the ON state) suggested that LDp/CDp may offer significant benefits over oral therapy regarding improvements in motor symptoms, sleep quality and overall quality of life [24]. These advantages were more pronounced in this younger subgroup than in the general study population, however, the potential implication that LDp/CDp initiation might be considered earlier in the disease stage [24] needs further study and is not covered by recent guidelines [7]. A very recent study showed that LDp/CDp may be promising for PD patients with relevant non-motor symptoms, as especially sleep, pain and constipation improved following LDp/CDp [25]. Furthermore, subcutaneous infusion can be helpful in patients with high-frequency oral levodopa medication and pronounced interaction with food intake. Clinical experience suggests that, among patients eligible for both subcutaneous infusion therapy and DBS, treatment choice is determined primarily by personal preference in most cases. In addition, magnetic resonance-guided focused ultrasound (MRgFUS) is another emerging treatment option [7]. Important clinical factors influencing this decision include clinical phenotype with medication refractory tremor (favouring DBS or MRgFUS, respectively), cognitive status (favouring pump therapy), the severity of peak-dose dopaminergic fluctuations and the presence of complications related to excessive dopaminergic stimulation (favouring DBS) [8]. Financial aspects should also not be completely disregarded when selecting patients for LDp/CDp [26, 27], as costs can be substantial. Ideal candidates typically present with a moderate levodopa equivalent daily dose—comparable to one cartridge of LDp/CDp (2,400/120 mg) per day—along with normal cognition or at most mild cognitive impairment and a stable caregiver environment. On the other hand, LDp/CDp is generally less appropriate for frail elderly patients with relevant cognitive decline or those lacking adequate support, as the risk of complications and poor adherence is higher in these patients. Caution is advised in patients with cognitive impairment and neuropsychiatric disorders (see chapter on side effects). Patients with a history of psychosis or those exhibiting ICD require a more detailed observation and monitoring as they are at a substantially higher risk of experiencing hallucinations or atypical psychosis [28, 29]. Therefore, a thorough risk-benefit assessment including a neuropsychiatric evaluation is needed before therapy is started.

In patients with dysphagia who also require enteral nutrition through a gastrostomy or jejunostomy tube, intestinal infusion of a levodopa/carbidopa gel may be preferred [30]. However, a recent preliminary study suggests that LDp/CDp could also lead to improvements in swallowing function [31]; while these findings are promising, further research is needed to better understand the long-term effects of LDp/CDp on dysphagia. Patients with a (very rare) levodopa/dopa-decarboxylase inhibitor associated microscopic colitis may benefit from LDp/CDp while bypassing the gastrointestinal tract [32].

No data are available regarding patients with frailty syndrome on LDp/CDp treatment. However, this patient group may also benefit from DAT as shown in a small study of advanced PD patients receiving intestinal levodopa-carbidopa-entacapone gel [33]. The authors believe that LDp/CDp initiation should be performed with caution in this subgroup due to the higher risk of neuropsychiatric side effects.

The findings are also relevant in clinical scenarios where oral administration is temporarily not possible, for example during a perioperative period or even in akinetic crisis. Managing PD during surgery can be difficult. Transdermal rotigotine has been used as a bridging strategy [34], but LDp/CDp may be an alternative. A recent case report described the successful use of this approach before surgery, showing its potential to ensure stable dopaminergic delivery when oral intake is restricted [35]. Additionally, LDp/CDp might be used as a rescue therapy in patients experiencing akinetic crises as recently demonstrated in two case reports. Labeit et al. described a 66-year-old patient who developed akinetic crisis following knee arthroplasty and omission of his established oral levodopa regimen. While attempts to reintroduce therapy via a nasogastric tube failed, LDp/CDp was initiated and the patient’s motor function improved within three days, and once swallowing recovered, oral therapy was resumed [36]. Similarly, another case report has shown LDp/CDp to be safe and effective in a patient with acute akinesia associated with dopamine-sensitive hydrocephalus [37]. These cases highlight the potential of LDp/CDp as an effective rescue strategy during akinetic crises. Another application might be the use of subcutaneous LDp/CDp as a bolus or short‑term infusion as an alternative to subcutaneous apomorphine when a standard oral levodopa challenge has failed and malabsorption is suspected, particularly in patients at risk for apomorphine.

### Expert recommendations

- The potential benefits of LDp/CDp initiation earlier in the PD stage (before the development of cognitive decline and frailty) should be considered. Ideal candidates for LDp/CDp therapy typically have a moderate levodopa equivalent daily dose (similar to one LDp/CDp cartridge, 2,400/120 mg), preserved cognitive function or only mild impairment and a stable caregiver environment.
- LDp/CDp is generally less appropriate for elderly patients with significant cognitive decline or inadequate support, as these factors increase the risk of complications and poor adherence.
- Patients with cognitive impairment, neuropsychiatric disorders or a history of psychosis and ICD require careful monitoring and a comprehensive risk–benefit assessment before initiating therapy.
- LDp/CDp may be considered in cases of temporary dysphagia such as in akinetic crisis or during surgery or in patients with persistent swallowing difficulties for oral medication.

## Initiation/dose determination

At the initiation of LDp/CDp therapy, the base infusion rate is typically calculated using a standardised conversion algorithm based on the patient’s total daytime levodopa dose from oral levodopa-containing medications and catechol O-methyltransferase (COMT) inhibitor over a 16-hour period. The resulting levodopa equivalent is then translated into a continuous 24-hour infusion rate using predefined dosing Table [38]. MAO-B inhibitors and other non-levodopa medications are generally not included in this calculation; however, if clinicians wish to account for them and aim to establish a LDp/CDp monotherapy, levodopa equivalent dose conversion tables are available [39]. However, in certain cases, especially when higher LDp/CDp doses are required, continuation or initiation of COMT inhibitors, especially opicapone or even add-on therapy with dopamine agonists (DA) or oral levodopa should be considered, if tolerable. This is particularly advisable when reducing the dosage of LDp/CDp is meaningful—e.g. in patients for whom a single LDp/CDp cartridge would otherwise not last 24 h (requiring cartridge changes at varying times of the day). The authors assume, based on clinical observations, that this approach may also be reasonable in those who develop skin reactions due to impaired absorption and/or the formation of fluid subcutaneous cushions [40].

The initiation of LDp/CDp infusion usually takes place while the patient is in the ON state. Although a loading dose (bolus) is an option if treatment is started in the OFF state [38], in the authors’ experience this is rarely done in routine care. Initiating therapy in the ON state is beneficial, as it takes approximately three to eight hours to reach steady-state plasma levels following the start of infusion depending on the infusion rate [41]. To ensure a smooth transition and prevent early OFF episodes, patients should continue their usual oral levodopa medication until LDp/CDp therapy begins. This approach improves patient acceptance and prevents a sudden worsening of symptoms at the start of treatment. In the authors’ clinical practice, MAO-B and COMT inhibitors are usually continued during LDp/CDp initiation (differing from earlier clinical trial protocols where COMT inhibitors were not permitted [14]) and often removed later. Furthermore, DA might be preserved at the beginning of LDp/CDp therapy, especially when higher dosages of DA are used to avoid DA withdrawal syndrome. Although, when starting with LDp/CDp, DA dosage might be reduced. Initiating LDp/CDp without prior medication adjustments allows for a more stable and patient-friendly transition. However, this approach may be adapted if there are adverse effects due to concomitant PD medications, particularly DA. In this case, the medication should gradually be tapered off over a period of a few weeks.

**Fig. 1:**
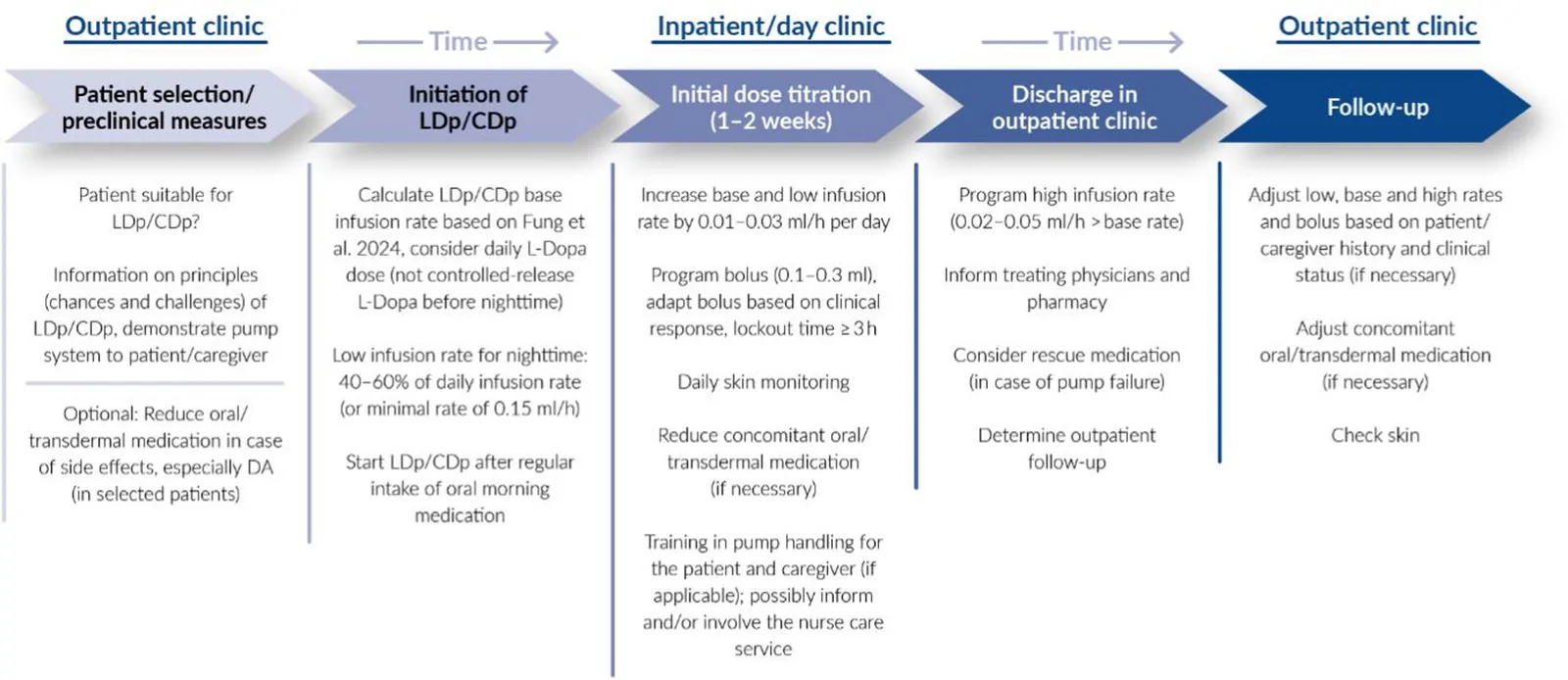
Management of LDp/CDp initiation in an inpatient and day-clinic setting. DA, dopamine agonist; Ldp/CDp, foslevodopa/foscarbidopa

The initial titration phase of subcutaneous LDp/CDp infusion therapy typically lasts about one to two weeks, while the subsequent optimization phase may extend over several weeks, since achievement of the maximum therapeutic benefit may require further dose adjustments according to the individual patient responsey(see Fig. 1). During this period, the base infusion rate is gradually adjusted, usually in increments of 0.01–0.03 ml/h per day, based on the patient’s clinical response. The goal is to achieve sustained ON time with minimal OFF episodes and minimal non-troublesome dyskinesias. As dosing is actively titrated during this phase, the prior exact conversion from oral levodopa is less critical. As with intestinal therapies, total daily doses often exceed those of oral regimens, since achieving equivalent symptom control with oral medications would require frequent dosing, which is not practical. Post-hoc analyses of a 52-week open-label study demonstrated that most study completers (63%) required increased doses for LDp/CDp optimisation after the initial conversion calculation [42]. Clinical experience shows that final infusion rates are often around 20% higher than the initially calculated base rate, as reported in several studies [28, 40, 43, 44], highlighting the importance of individualised titration for optimal outcomes and the option to continue other medication (COMT inhibitors, DA or oral levodopa).

Nighttime dopaminergic requirements are lower than during the day, likely due to circadian rhythm influences. Typical nighttime rates range between 40 and 60% of the daytime rate or the minimal possible rate (0.15 mL/h). However, clinical experience has shown considerable interindividual variability: while some patients benefit from a high nighttime rate (e.g. 80% of the daytime rate), others may experience adverse effects such as hallucinations, therefore needing a significantly reduced dose [28, 45]. In a few cases, based on the authors’ clinical experience, even complete discontinuation of nighttime infusion was considered. However, if the infusion is paused at night, a new cannula and glass vial must be used each morning, which increases patient burden and treatment costs and a loading dose in form of a bolus.

The possibility of the ‘high infusion rate’ is generally not used during the initiation phase, unless it becomes clear that certain times of the day, i.e. the afternoon or during special physical activity (change to high rate 1–2 h before expected effect), require increased dopaminergic support. This need can often be inferred indirectly from the frequency of patient-initiated extra or higher doses and/or additional rescue medication.

The availability of an extra dose of LDp/CDp plays an important role by providing the flexibility needed to manage day-to-day symptom variability and acute OFF episodes. It is usually taken by the patients as the first option before other rescue interventions; however, its onset of action may be too slow in some patients (usually 60–90 min). Pharmacokinetic data show that peak levodopa concentrations following an LDp/CDp dose are reached later compared to oral levodopa in combination with carbidopa or benserazide, inhaled levodopa or subcutaneous/sublingual apomorphine [41, 46]. This difference in onset should be considered when planning individualised add-on rescue strategies.

After discharge from an inpatient setting, patients are usually scheduled for outpatient or day-clinic follow-up appointments at the treatment centre. These visits allow infusion parameters to be adjusted and therapy to be optimised according to each patient’s individual response. At this point, the programming of a ‘high infusion rate’ is useful (see Fig. 1).

### Expert recommendations

- Therapy initiation should start during the ON phase. To prevent early OFF episodes, patients should continue their usual oral levodopa medication until LDp/CDp therapy is initiated. This approach improves patient acceptance and prevents a sudden worsening of symptoms at the start of treatment.
- Concomitant PD medications, including DA, MAO-B inhibitors and COMT inhibitors, can usually be continued during LDp/CDp initiation. This approach may be adapted if there are adverse effects from the concomitant PD medications. In this case, the medication should gradually be tapered off slowly over a period of a few weeks before initiation of LDp/CDp.
- The initial titration phase for LDp/CDp typically lasts around one week, involving daily rate adjustments of 0.01–0.03 mL/h based on the patient’s clinical response. Final doses often exceed initial estimates by around 20%, highlighting the importance of individualised titration rather than relying solely on the conversion.
- Typical nighttime rates range between 40 and 60% of the daytime rate or the minimal possible rate (0.15 mL/h) but can be higher, lower or even be omitted. If infusion is paused at night, a new cannula and glass vial must be used each morning.

## Monotherapy/concomitant medication

Study data show that both LDp/CDp monotherapy and combination therapy are valid options. Early trials evaluated LDp/CDp as monotherapy without oral rescue therapy [41], while later studies allowed concomitant PD medications (excluding oral levodopa and COMT inhibitors) and reported no additional safety concerns [14]. A recent post-hoc analysis of three trials showed that monotherapy and combination therapy achieved similar results, including significant reductions in OFF time and improvements in ON time without troublesome dyskinesia so that monotherapy was sustained for up to 96 weeks in a portion of patients [15]. However, a small single-centre retrospective study suggested that monotherapy might increase the likelihood of treatment discontinuation [47]. Whether monotherapy should be the preferred approach remains therefore debated. While some experts support a gradual reduction of concomitant medications towards monotherapy to minimise polypharmacy, others favour combination therapy, often with COMT inhibitors and DA (usually at a lower dose than before), to optimise tolerability and reduce infusion-related side effects and/or treatment costs. In practice, achieving monotherapy can be challenging, particularly in outpatient settings where existing medications are often maintained.

### Expert recommendations

- Both LDp/CDp monotherapy and combination therapy are valid options; monotherapy is not the primary goal.
- Monotherapy should not be pursued too early but rather introduced gradually to avoid psychotropic complications. Ultimately, treatment decisions should be made on an individual basis, considering the patient’s clinical presentation and tolerability.

## Side effects and their management

Safety data from clinical trials showed that LDp/CDp was well tolerated and adverse events indeed frequent, but generally mild to moderate [13, 41]. Infusion site reactions and hallucinations are probably the most common and clinically relevant side effects, as demonstrated by post-marketing safety data [48].

### Skin reactions

In both clinical practice and clinical trials, skin reactions were found to be the most frequent adverse events after 12 [13], 52 [14] and 96 weeks [15]. This finding is not surprising given the subcutaneous route of administration and the similar occurrence observed with other subcutaneous therapies, such as apomorphine [49]. Interestingly, clinical experience suggests that the frequency and severity of skin reactions tend to decrease over time, a trend also reported in clinical studies [15]. This improvement is likely due to strict adherence to essential hygienic practices [38, 50].

These skin reactions primarily indicate aseptic inflammation of the subcutaneous tissue [51, 52], rendering antibiotic therapy generally unnecessary. In general, appropriate disinfection and hygiene requirements are mandatory for reducing the risk of skin reactions. Experience has shown that early intervention using topical corticosteroids (e.g. mometasone 0.1%) can be effective, either as prophylactic treatment for selected patients with early skin reactions or as an intervention when symptoms first appear [10, 50]. Systemic antibiotic therapy should only be considered if symptoms worsen despite topical treatment, or if systemic signs of inflammation develop, such as fever, leucocytosis or elevation in C-reactive protein (CRP) levels [10]. Surgical interventions are rarely necessary, reflecting improved management strategies and preventive measures. See Table 1 for practical measures to reduce skin reactions.

**Table 1:** Practical measures to reduce skin reactions

Measure	Rationale
Regular hygiene training for patients and caregivers	Essential for preventing complications, especially after caregiver changes
Removal of the cannula two to three hours after stop of infusion	Reduces local irritation, use of topic corticosteroid due to reduction of local inflammation
Daily needle change	Reduces local irritation, risk of inflammation and occurrence of nodules
Consider acid–base balance diet and reduction of stress	Physiological stress and pH shifts may exacerbate reactions
Reduce dose and infusion volume (by adding opicapone or DA)	Skin reactions may be dose-dependent; adjusting these can reduce severity

### Hallucinations/atypical psychosis

Although LDp/CDp is a subcutaneous continuous formulation of levodopa with assumably lower/no peak dosages, clinical trials consistently show a higher risk of hallucinations compared with oral levodopa therapy, with rates ranging from 0% to 18.5% versus 0% to 2.2%, respectively [15]. These observations have been confirmed in clinical practice and may be even more frequent or more pronounced, as shown by a recent study [43]. In a vulnerable real-world PD population neuropsychiatric and/or cognitive worsening under LDp/CDp was more frequent compared to the clinical trials (47% vs. 7%–17%) but was generally mild, especially when clozapine was used proactively [43].

Poor cognitive status, advanced age and a history of hallucinations appear to be potential predictors for hallucinations under LDp/CDp [28]. Nevertheless, several centres have reported successful initiation of LDp/CDp in patients with a history of hallucinations, provided close monitoring is ensured during the transition phase. Consequently, hallucinations are not considered to be an absolute contraindication. Hallucinations can be often managed by lowering the nighttime infusion rate, reducing concomitant oral dopaminergic therapy such as DA [53], discontinuation of cholinesterase inhibitors in case of cognitive decline, adding rivastigmine in case of relevant cognitive deficits and/or adding quetiapine or better clozapine if hallucinations persist.

Although cognitive impairment and advanced age are well-recognised risk factors for typical psychosis, particularly visual hallucinations, recent evidence from Japan indicates that patients who develop atypical psychosis exhibit a different profile. This form of psychosis is characterised by delusions resembling those of mania or schizophrenia and auditory or somatic hallucinations. The study indicates that individuals exhibiting ICD prior to starting LDp/CDp may be at increased risk of atypical psychosis.

Another study has shown that ICD may even worsen after switching to LDp/LDp [54]. Weight loss following initiation of LDp/CDp may be an additional persistent risk factor for the development of psychosis [44]. Rather than excluding these patients, they should be monitored closely, and a management strategy needs to be in place to address early signs of psychosis [29].

### Expert recommendations

- Should initial skin reactions occur, it is recommended to reduce needle retention time to one or two days and to reduce medication dosage (e.g. drug holiday for some hours or a day with oral levodopa).
- Early use of topical corticosteroids (e.g. mometasone 0.1%) can be effective, either as prophylactic treatment or as an intervention when symptoms first appear.
- Hallucinations as side effects are quite frequent but can be managed by lowering the nighttime infusion rate and/or reducing concomitant oral dopaminergic therapy such as DA, MAO-B inhibitors or opicapone.
- Patients with pre-existing ICD may be at an increased risk of developing atypical psychosis when starting LDp/CDp therapy. It is therefore recommended to implement close monitoring and proactive management of the early symptoms of psychosis.

## Discontinuation

**Fig. 2:**
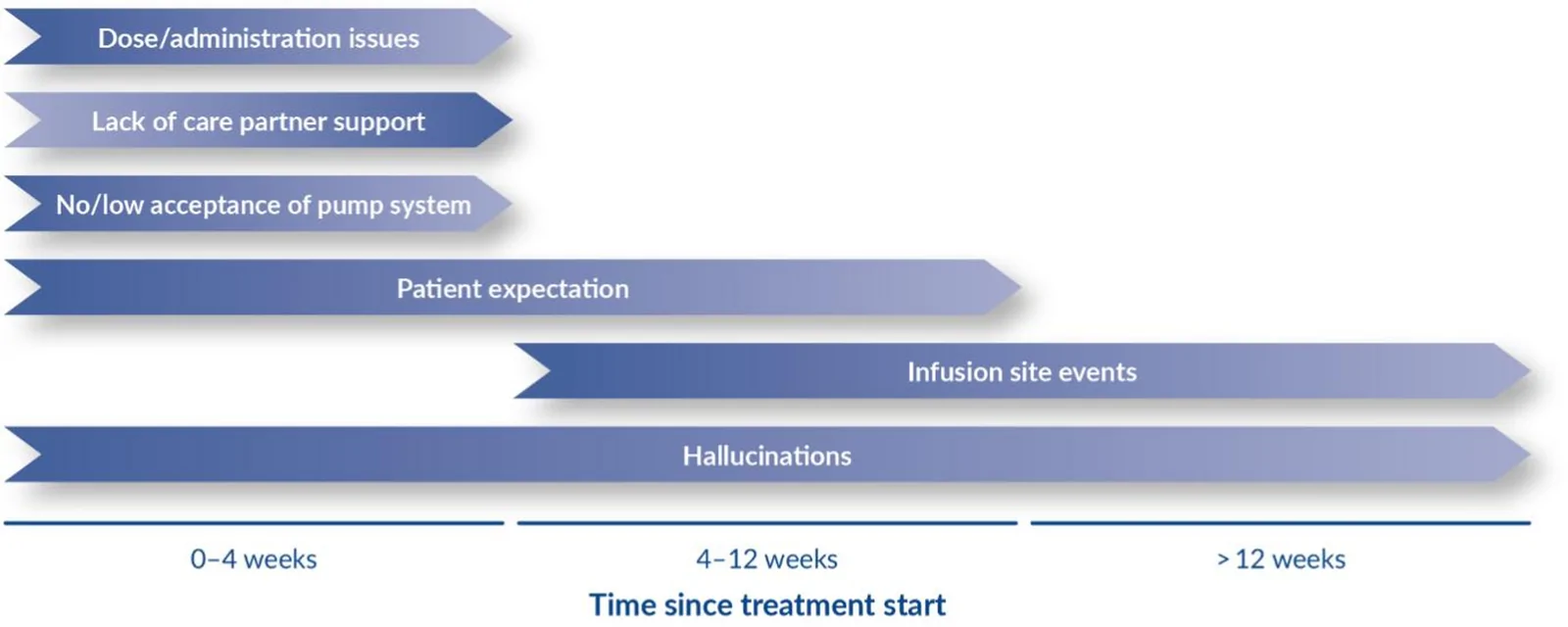
Reasons and typical time period of discontinuation

Despite careful patient selection, the discontinuation rate for subcutaneous LDp/CDp pump therapy in Germany is currently around 35% [55], which is higher than that observed with intestinal pumps or DBS. In the long term, discontinuation rates appear comparable across subcutaneous modalities, with similar rates observed for continuous subcutaneous apomorphine infusion [56, 57]. In a recent study from Japan, where LDp/CDp was introduced around four months earlier, the dropout rate was even higher (around 40%) [58]. Discontinuation mainly occurred within the first four to twelve weeks of treatment. The reasons for this high dropout rate are diverse and range from unmet expectations, insufficient efficacy and adverse effects to a lack of home care support [58]. However, the reasons for discontinuation seem to change over time (Fig. 2) [59].

We believe that one major contributor to the high discontinuation rate may be the lower threshold for initiating therapy compared to intestinal pumps and DBS. While its flexibility improves accessibility, it also increases the likelihood of early discontinuation, especially when patients perceive the pump as subjectively too burdensome. Importantly, many patients who stop treatment may later resume it when their condition worsens or the side effect disappeared (e.g. psychosis), making such interruptions relatively manageable. Most experts agree that continuous therapy should ideally be started early during advanced PD. However, the risk-benefit balance of early use remains uncertain. We speculate that while delaying initiation may reduce discontinuation rates, it may also diminish the potential advantages of early intervention. However, this hypothesis requires validation in appropriate clinical studies.

Adverse effects can lead to treatment discontinuation. Skin reactions, which typically occur in the first 12 weeks, remain a contributing factor but tend to decrease over time as management strategies improve. On the other hand, hallucinations and psychosis remain clinically relevant reasons for discontinuation.

A real lack of response to subcutaneous pump therapy is rare and usually does not reflect the efficacy of LDp/CDp, but rather fluctuations. Subcutaneous absorption can vary individually, and protein interactions in the bloodstream or the ‘blood-brain barrier’, especially after meals, may contribute to OFF phases. In most cases, ‘lack of efficacy’ is due to underdosing or depot formation, which can often be resolved by adjusting needle placement. Recently published data [47] suggest that treatment optimisation strategies may also play a role. Patients who continued LDp/CDp therapy often benefited from flexible dose adjustment protocols, including nighttime tapering and the use of oral concomitant medication. By contrast, patients managed under a rigid monotherapy approach were more likely to discontinue treatment. Interestingly, despite having higher average doses and elevated plasma concentrations, the discontinuation group did not experience an improvement in their motor symptoms. This observation suggests that non-dopaminergic mechanisms may contribute to OFF periods, which do not always respond to increased dopaminergic stimulation. Unrealistic expectations for rapid and complete symptom relief may have driven premature discontinuation before therapeutic optimisation could be achieved [47]. The reason may be the absence of an immediate ‘ON-kick’ effect, which patients often associate with oral levodopa medications. This lack of fast symptom relief can lead to a subjective perception of reduced benefit. It is therefore critical that patients are informed about both the benefits and practical challenges of LDp/CDp therapy. Setting realistic expectations is essential, as dose optimisation and routine adjustment take time. Clear instructions, training and regular practice help ensure adherence, long-term success and satisfaction [60]. Both the support of caregivers and professional care support are important. The latter is particularly crucial with regards to providing education on device handling and troubleshooting. Encouragingly, discontinuation rates have declined with growing clinical expertise, including improved skin management, more rigorous patient selection and structured patient training.

### Expert recommendations

- ‘Lack of efficacy’ is often due to underdosing or depot formation, which can often be resolved by adjusting needle placement.
- Patients should be given realistic expectations, as dose optimisation and routine adjustments take time.
- Education and training of pump handling of both patients and caregivers with support from a healthcare team is recommended.

## Practical implementation

### Settings

The subcutaneous infusion of LDp/CDp can be initiated and titrated in various clinical settings, including inpatient units, outpatient clinics and day-clinics. Real-world study data have shown that inpatient setting [61], as well as outpatient initiation followed by visits from specialist nurses over several weeks [62], and more intensive day-clinic protocols involving five full-day clinic visits within three weeks after initiation [19] are all feasible and safe. Even though neuropsychiatric complications occurred more often in the cohort with outpatient initiation, discontinuation rates remained similar across the different settings. However, the authors view the initiation of LDp/CDp in an outpatient clinic setting very critically compared with inpatient or day-clinic settings. At the end, the choice of setting should be made on an individual basis, looking at patient characteristics and wishes, available resources and the local healthcare context [63].

Follow-up outpatient care is important for the long-term success of LDp/CDp. After initial pump fitting, however, ongoing care is often insufficient, which can be a reason for treatment discontinuation. To ensure optimal outcomes, it is recommended to confirm that the patient’s general practitioner/treating practice neurologist is familiar with the pump system and supports its use. Close collaboration between specialised centres and primary care providers is important to maintain treatment adherence and manage complications.

### Switching from other DAT

Real-world evidence shows that it is feasible, effective and safe to change from continuous subcutaneous apomorphine infusion to LDp/CDp. It has been shown that three months after the change, motor complications were significantly reduced, with improvements in functional scores, quality of life, clinical global impression of severity and neuropsychiatric inventory. However, behavioural changes (apathy) were observed in two of the 14 patients highlighting the importance of clinical awareness for these changes [64]. Similarly, a case series has shown that transitioning from intestinal levodopa/carbidopa to LDp/CDp is possible and safe and associated with modest improvements in PD symptoms [65]. While these data are limited, they suggest that LDp/CDp is an effective and well-tolerated second-line infusion option in clinical practice when prior DAT are insufficient. However, a recent case report described a patient with advanced PD who experienced a marked deterioration in motor function (lack of therapeutic response) following the switch from intestinal levodopa/carbidopa to LDp/CDp [66]. More data is therefore needed.

### Expert recommendations

- LDp/CDp can be safely initiated in inpatient units or day clinics. The choice should consider patient characteristics, preferences, available resources and local healthcare context.
- Long-term success depends on consistent outpatient care following the initiating of the pump. It is recommended that the patient’s treating neurologist is familiar with the pump system and supports its use. The general practitioner should be aware and capable of handling skin reactions and check vitamin B12 and B6 levels. Close collaboration between specialised centres and primary care providers is essential to maintain adherence and manage complications.
- Changing to LDp/CDp is usually conducted using an overlapping approach and is generally straightforward. It is recommended that existing access points (e.g. percutaneous endoscopic gastronomy tubes) remain in place until the effectiveness of LDp/CDp has been confirmed.

## Open questions and future directions

Several open questions remain regarding LDp/CDp therapy, also because long-term experience (> 24 months) is lacking. Systematic analyses of real-world data, especially on neuropsychiatric symptoms, and international comparisons are needed to clarify regional differences. Dose conversion strategies require re-evaluation and possible refinement by integrating patient-specific factors such as adipose tissue distribution. Further studies should assess effects of vitamin B12 levels, gastrointestinal function (especially dysphagia) and skin reactions, including potential predictive markers and management options. The efficacy and cost-effectiveness of COMT and MAO-B inhibitors in subgroups, as well as the combined use of deep brain stimulation and LDp/CDp, warrant further investigation. Finally, further retrospective as well as prospective analyses of adherence and discontinuation rates in experienced centres are needed to guide clinical practice.

The key priorities for advancing subcutaneous pump therapy are to reduce device size and weight [60], improve durability and enhance usability by providing clearer alarm sounds (especially during the night), enabling data storage and export, adjusting flow automatically and offering app-based remote control for healthcare professionals. Long-term developments may involve an adaptive pump system that adjusts delivery based on data provided by wearables or biomarkers. Another potential innovation is a patch-pump system analogous to insulin therapy.

While fully closed-loop systems are currently unrealistic due to the lack of a clear control variable, very delayed resorption and steady state characteristic, the limited interpretability of continuous levodopa monitoring as well as pragmatic improvements, such as circadian flow-rate programming, could help to optimise management of predictable OFF phases. Individualised profiles derived from multi-day data and sensor-based feedback could help to prevent major symptom fluctuations.

At the moment, only one subcutaneous levodopa/carbidopa formulation is available to treat advanced PD. However, another formulation (ND0612) has been developed [67]. A recent meta-analysis of five studies showed that ND0612 significantly reduced OFF time and improved quality of life among patients with advanced PD [68]. In a recent review, a retrospective comparative data analysis of the two subcutaneous levodopa formulations has been performed [69]. Both systems have their advantages and limitations, which will become clearer as ND0612 is introduced into clinical practice. Long-term data on the efficacy and safety of both formulations are needed. As more device-assisted levodopa treatments become available, selecting the optimal therapy for advanced PD will require an individualised approach.

## Conclusions

Treatment with LDp/CDp for advanced PD has been shown to be highly effective in managing motor fluctuations and provides stable, sustained clinical benefits [70]. This manuscript adds practical insights that support the implementation, optimisation and management of LDp/CDp therapy in routine care by incorporating real-world clinical experience. Concomitant oral medication should be adjusted cautiously, as monotherapy is not the primary goal. Dermatological reactions are common but can be effectively managed in most cases with appropriate strategies such as reduction of needle retention time, medication dosage and thorough hygiene. Elderly patients with cognitive impairment or patients with a prior history of hallucinations require particular attention regarding neuropsychiatric adverse effects, particularly hallucinations. Clinical experience over the last two years led to reduced rates of early therapy discontinuation, which were initially more prevalent. However, comprehensive education of both patients and caregivers remains important for successful treatment. Overall, therapy with LDp/CDp has demonstrated a consistent therapeutic effect and favourable tolerability, and no previously unrecognised safety concerns have been identified during routine clinical use.

## Data Availability

No datasets were generated or analysed during the current study.
